# The effectiveness and safety of anti-fibrinolytics in patients with acute intracranial haemorrhage: statistical analysis plan for an individual patient data meta-analysis

**DOI:** 10.12688/wellcomeopenres.13262.3

**Published:** 2019-06-11

**Authors:** Katharine Ker, David Prieto-Merino, Nikola Sprigg, Abda Mahmood, Philip Bath, Zhe Kang Law, Katie Flaherty, Ian Roberts

**Affiliations:** 1 Clinical Trials Unit, London School of Hygiene & Tropical Medicine, London, UK; 2 Catholic University of Murcia, Campus de los Jeronimos, Murcia, Spain; 3 London School of Hygiene & Tropical Medicine, London, UK; 4 Stroke, Division of Clinical Neurosciences, Faculty of Medicine & Health Sciences, Clinical Sciences Building, Nottingham City Hospital, Nottingham, UK

**Keywords:** antifibrinolytics, meta-analysis, trials

## Abstract

Abstract

Introduction: The Anti-fibrinolytics Trialists Collaboration aims to increase knowledge about the effectiveness and safety of anti-fibrinolytic treatment by conducting individual patient data (IPD) meta-analyses of randomised trials. This article presents the statistical analysis plan for an IPD meta-analysis of the effects of anti-fibrinolytics for acute intracranial haemorrhage.

Methods: The protocol for the IPD meta-analysis has been registered with PROSPERO (CRD42019128260). We will conduct an individual patient data meta-analysis of randomised controlled trials with 500 patients or more assessing the effects of anti-fibrinolytics in acute intracranial haemorrhage. The primary outcomes will be 1) death from stroke or head injury within 30 days of randomisation, and 2) death from stroke or head injury, or dependency within 90 days of randomisation. The primary outcomes will be limited to patients treated within three hours of injury or stroke onset. We will report treatment effects using odds ratios and 95% confidence intervals. We use logistic regression models to examine how the effect of anti-fibrinolytics vary by time to treatment, severity of intracranial bleeding, and age. We will also examine the effect of anti-fibrinolytics on secondary outcomes including death, dependency, vascular occlusive events, seizures, and neurological outcomes. Secondary outcomes will be assessed in all patients irrespective of time of treatment. All analyses will be conducted on an intention-to-treat basis.

Conclusions: This IPD meta-analysis will examine important clinical questions about the effects of anti-fibrinolytic treatment in patients with intracranial haemorrhage that cannot be answered using aggregate data. With IPD we can examine how effects vary by time to treatment, bleeding severity, and age, to gain better understanding of the balance of benefit and harms on which to base recommendations for practice.

## Introduction

Traumatic and spontaneous intracranial bleeding are leading causes of death and disability worldwide. Traumatic brain injury, responsible for over 10 million deaths or hospitalisations each year ^1^, is often accompanied by intracranial bleeding and the larger the bleed the worse the outcome ^2^. Bleeding continues after hospital admission in most patients with moderate or severe traumatic brain injuries ^3^. Haemorrhagic stroke affects about six million people every year worldwide ^4^. About three million die and many survivors are permanently disabled ^4^. Once again, bleeding can continue for up to 24 hours after stroke onset, although is most common in the first few hours ^5^. The continuation of bleeding in the hours after onset in both traumatic and spontaneous intracranial bleeding, offers a therapeutic window to reduce the extent of the bleeding and improve patient outcomes.

Anti-fibrinolytics reduce bleeding by inhibiting the enzymatic breakdown of fibrin blood clots. They reduce surgical bleeding by about one third, irrespective of the site of surgery ^6, 7^. When given within three hours of onset, the anti-fibrinolytic tranexamic acid (TXA) reduces death due to bleeding in trauma and postpartum haemorrhage, with no evidence of heterogeneity by type of bleeding ^8^. However, in both trauma and postpartum haemorrhage there is no apparent benefit when treatment starts more than three hours after bleeding onset. TXA does not appear to increase the risk of thromboembolic events in extracranial bleeding ^7– 9^.

The improved outcomes with anti-fibrinolytic treatment in extracranial bleeding raises the possibility that they might improve outcomes after intracranial bleeding. There have been two small trials of TXA in traumatic brain injury ^10, 11^; both recruited patients within eight hours of injury. A meta-analysis showed a significant reduction in haemorrhage expansion with TXA ^12^. However, even when combined the trials are too small to determine the overall risks and benefits, and whether these vary with treatment delay. Larger trials are ongoing. Trials of TXA in aneurysmal subarachnoid haemorrhage show less re-bleeding but more ischemia ^13^. However, the long courses of treatment in these trials, unlike the eight-hour courses used in extracranial bleeding, may account for the increase in ischaemia. Larger trials of shorter regimens are underway ^14, 15^.

The Anti-fibrinolytics Trialists Collaboration (ATC) aims to increase knowledge about the effectiveness and safety of anti-fibrinolytic treatment by conducting individual patient data (IPD) meta-analyses of randomised trials of anti-fibrinolytics in acute severe bleeding involving 500 patients or more. This article presents the statistical analysis plan for an IPD meta-analysis of the effects of anti-fibrinolytics in acute intracranial haemorrhage. We are currently aware of four trials of TXA in patients with intracranial haemorrhage that meet the inclusion criteria for the IPD, the CRASH-3, TICH-2, ROC TXA and ULTRA clinical trials ^14– 17^. The lead investigators of the CRASH-3 and TICH-2 trials are members of the ATC and co-authors of this statistical analysis plan. The lead investigators of the ROC TXA and ULTRA trial will also be invited to join the ATC and submit IPD for analysis. Versions 1 and 2 of this plan were prepared prior to knowledge of the results of any of these trials. However, revisions for the preparation of version 3 were made after the results of the TICH-2 and ROC TXA trials were publically available.

## Methods

### Identification of eligible trials

We will conduct an individual patient data meta-analysis of randomised controlled trials with 500 patients or more that assessed the effects of anti-fibrinolytics (aprotinin, tranexamic acid, epsilon-aminocaproic acid and p-aminomethylbenzoic acid) in acute intracranial haemorrhage. To be included, a randomised trial must: i) be prospectively registered (i.e. before the first participant is enrolled) in a trial registry; ii) randomise 500 patients or more; iii) be judged to be at low risk of bias for sequence generation, allocation concealment and blinding of outcome assessment. We will identify trials from a register of anti-fibrinolytic trials maintained by the LSHTM Clinical Trials Unit. Records included in this register are identified by running regular searches of the following databases: MEDLINE, EMBASE, the Cochrane Central Register of Controlled Trials (CENTRAL), Database of Research in Stroke (DORIS), Web of Science, PubMed, Popline and the WHO International Clinical Trials Registry Platform. We will screen abstracts for relevant trials and apply the relevant selection criteria. We discuss reasons for exclusion and resolve discrepancies by consensus. Two reviewers will extract data to minimise bias. We will extract and describe data on patients and interventions for all trials irrespective of sample size. However, only IPD from trials involving 500 patients or more will be sought and included in the analysis to minimise small study effects. We will analyse individual patient data for baseline, outcome, and predictor variables; dates and times of randomisation and death. We registered the protocol in November 2016 (CRD42016052155) without any knowledge of the results of the large ongoing trials. The registration record has since been superseded by a new record registered in April 2019 (CRD42019128260) which reflects revisions to the inclusion criteria. We judge that separate institutional review board (IRB) approval for this study is not required. This project involves the analysis of existing trial data. Each trial providing individual patient data will have received local ethical approval. The planned study will not require further recruitment or data collection from patients and the analysis will not include identifiable data. The lead investigators of the CRASH-3 and TICH-2 trials agree that use of the IPD data from their trials does not require separate ethics committee approval. If, however, there is uncertainty about the use of data from other eligible trials, we will seek approval from the IRB board that originally approved the trial before including the data in the analysis.

### Comparison of baseline measures between trials

Before conducting analyses to estimate the effects of anti-fibrinolytic treatment, we will present descriptive analyses to show any differences in baseline characteristics between the types of patient enrolled in the included trials. We will present statistical comparisons of baseline means ( *t*-tests) and prevalence measures (chi-squared tests) for patients enrolled in the included trials.

### Intention to treat analyses and missing data

We aim to include all randomised patients, regardless of whether they received the trial treatment, on an intention-to-treat basis. For patients who withdraw consent after randomisation, data collected up to the point of withdrawal will be included. We do not anticipate substantial amounts of missing data for the primary outcomes and subgroup factors. However, in the event that missing data is significant we will use a range of statistical approaches and will assess the impact of missing data on the results by conducting sensitivity analyses. We do anticipate substantial missing data for neuro-radiological outcomes measures, since many patients will not be scanned before and/or after randomisation because they died or did not require re-scanning. Indeed, the pilot data from the CRASH-3 Intracranial Bleeding Mechanistic Sub-study suggests that post-randomisation scans are less likely to be done in patients who die soon after admission (i.e. patients with a low Glasgow Coma Scale score) but also in patients who have a mild head injury (i.e. patients with a high Glasgow Coma Scale score) who do not need a second scan. We will report the number of patients without pre- and post-randomisation scans by treatment arm. If the outcome of interest (haemorrhage expansion) is associated with the reason the data are missing (for example, patients with haemorrhage expansion may be more likely to die before the second scan), imbalance in missing data by treatment group could cause bias. If we suspect data are missing not at random, we will assess the impact of this in sensitivity analysis.

### Primary outcomes

There are two primary outcomes.

1) Death from stroke or head injury within 30 days of randomisation among patients treated within three hours of injury or stroke onset.

2) Death from stroke or head injury, or dependency at final follow-up within 90 days of randomisation among patients treated within three hours of injury or stroke onset.

The eligible trials identified to date use the modified Rankin scale or Disability Rating scale to assess dependency. Dependency will be defined as a score of 4–6 on the modified Rankin scale or a score of ≥12 on the Disability Rating Scale.

Although some trials recruit patients up to eight hours after injury or stroke onset, evidence from pathophysiological studies and trials of TXA in extra-cranial bleeding strongly suggest that treatment beyond three hours of onset is unlikely to improve outcomes. We believe that this is even more likely in the context of intracranial bleeding because the majority of bleeding occurs within the first few hours of injury ^18^. We will examine the effects of anti-fibrinolytics on death using logistic regression. We will report treatment effects using odds ratios (OR) and 95% confidence intervals (95% CI). We will first assess the homogeneity of the treatment effects between trials by estimating a random effects model where both the intercept and the treatment effect will be allowed to have a distribution across trials. The variance of the distribution of the treatment effect will give us an idea of the heterogeneity between trials. However, if only very few trials are included in the meta-analysis, instead of a random effects model we will examine the heterogeneity by including an interaction term between the treatment and the trial variable and reporting the p-value.

We will also plot a Kaplan-Meier curve for survival analysis comparing outcome of patients in treatment and placebo arms.

### Subgroup analyses for primary outcomes

(a) Time to treatment – Does treatment delay modify the proportional effect of anti-fibrinolytics on death and or dependency taking into account any other independent relationships between severity/age and the treatment effect?

We define treatment delay as the time from injury or symptom onset to randomisation. We appreciate that there will be some time interval between randomisation and treatment delivery but not all trials record the time of treatment delivery and we expect this interval to be short (0–15 minutes). We expect that the effect of TXA will vary by time to treatment with early treatment being most effective. Initially, we will plot treatment effects and 95% confidence intervals by 60-minute intervals of treatment delay. In addition, we will assess the impact of treatment delay on treatment effect in a regression analysis that includes terms for hours of treatment delay and its square (because of potential non-linearity of the treatment effect), and interactions between these two variables with treatment group. To explore the interaction between treatment effect and time, we will use the data on all treated patients and not only those treated within three hours.

We will check for potential heterogeneity of these effects across trials, by running a random effects models allowing the coefficients to vary randomly across trials. However, if we only include a small number of trials, instead of the random effects model we will include a triple interaction between the terms for treatment delay, the treatment group, and the trial.

Because severity of intracranial bleeding and age could confound impact of treatment delay on treatment effectiveness, we will control all models for GCS and age (10-year intervals) which are strong risk factors for death. If the above regression analyses indicate a trend towards decreasing treatment effectiveness with increasing delay, we will estimate the time at which the estimated odds ratio reaches the null (1.00) and the time at which the lower 95% confidence interval reaches the null.

Because there is strong prior evidence to expect a time to treatment interaction, two-way interaction tests will be regarded as statistically significant and thus providing evidence of effect modification if the two-sided P-value is less than 0.05.

Assessment of regression dilution bias: Because time of bleeding onset (i.e. time of injury or stroke onset) is often uncertain, measurement error is inevitable. We will investigate the impact of misclassification of treatment delay in sensitivity analyses using a range of plausible errors. We will add a random number of minutes to the treatment delay using a uniform distribution with a constant minimum set at 0 and four sets of maximum value: 15, 30, 45 and 60 minutes. The corrections are based on data from an audit of treatment delay in a large clinical trial in traumatic brain injury (the CRASH-3 trial) in which treatment delay was rarely over-estimated but often under-estimated (mean under-estimation 51 minutes). For each of the four maximum values, we will re-estimate the final model 100 times to obtain ranges for the time to treatment interaction.

(b) Severity of intracranial bleeding – Does severity modify the proportional effect of anti-fibrinolytics taking into account any other independent relationships treatment delay or age and treatment effect?

We will examine the effect of anti-fibrinolytics stratified by baseline severity. All the eligible trials identified to date record GCS at baseline. We will examine three subgroups based on baseline GCS: mild (GCS 13-15), moderate (GCS 9-12) and severe (GCS 3-8). We will use interaction tests to see whether the effect of the treatment (if any) differs across these subgroups. We will also assess the impact of baseline severity on the treatment effect in a regression analysis that includes continuous terms for severity and its square (because of potential non-linearity of the treatment effect). Because treatment delay and age could confound impact of severity on treatment effectiveness, we will control all models for treatment delay and age (10-year intervals) and their interaction with treatment. Unless there is strong evidence against the null hypothesis of homogeneity of effects (i.e. p<0.01) the overall odds ratio will be considered the most reliable guide to the approximate treatment effect in all patients.

(c) Age – Does the patient’s age modify the proportional effect of anti-fibrinolytics taking into account any other independent relationships with treatment delay or severity and the treatment effect?

We do not expect the proportional benefits of anti-fibrinolytics to reduce with increasing patient age. However, because traumatic and spontaneous intracranial bleeding are increasingly common in older patients, who are sometimes denied potentially effective treatments on the basis that there is insufficient evidence in older patients, it will be important to consider this question. We will therefore conduct regression analyses to assess the impact of age on the treatment effect in a regression analysis that includes continuous terms for age and its square (because of potential non-linearity of the treatment effect) and their interaction with treatment. Because treatment delay and severity could confound the effect of age on treatment effectiveness, we will control all models for treatment delay and severity and their interactions with treatment. Unless there is strong evidence against the null hypothesis of homogeneity (i.e. p<0.01), the overall odds ratio will be considered the most reliable guide to the approximate treatment effect in all patients.

### Secondary outcomes

We will assess the effect of TXA on the following secondary outcomes in all patients, irrespective of time of treatment.

Clinical outcomes

- Death within seven days of randomisation
- Dependency score
- Cause specific mortality
- Vascular occlusive events (myocardial infarction, stroke, deep vein thrombosis, pulmonary embolism)
- Seizures

Neuro-radiological outcomes

The key neuro-radiological outcome will be the total volume of intracranial bleeding after randomisation (adjusting for total volume of intracranial bleeding at baseline if baseline volume is available).

Other neuro-radiological outcomes will be;

- New focal ischaemic lesions (ischaemic lesions which appear on a post-randomisation scan but not known to be present pre-randomisation scan)
- Frequency of progressive haemorrhage (number of patients with a post-randomisation CT scan with total haemorrhage volume of more than 33% of the volume on the pre-randomisation scan)
- The total volume of intracranial bleeding after neurosurgery (accounting for total volume of intracranial bleeding at baseline if baseline volume is available)

A selection of approximately 1750 patients in the CRASH-3 trial are included in a neuro-radiological sub-study in which a simple validated rating scale (ABC/2) is used to measure intracranial haemorrhage. All patients in the TICH-2 trial undergo brain imaging before and the majority do so again after randomisation. Patients in the ROC TXA trial undergo CT scans if clinically indicated. Both the TICH-2 and ROC TXA trials also use the ABC/2 method to estimate intracranial haemorrhage.

Subgroup analysis of neuro-radiological outcome

We will conduct subgroup analysis to examine whether the effect of TXA on the key neuro-radiological outcome of total volume of intracranial bleeding after randomisation varies by haematoma type (intra-parenchymal, intra-ventricular, epidural, subdural, subarachnoid, lobar, deep). Intra-parenchymal haemorrhage is at the greatest risk of expansion and we hypothesise that TXA will be most likely to reduce bleeding of this type. Intra-ventricular haemorrhage and clots could block the flow of cerebrospinal fluid and thereby increase the risk of hydrocephalus. TXA therefore may be ineffective or even harmful for patients with this type of bleeding. We will use interaction tests to see whether the effect of TXA (if any) differs across these subgroups. Unless there is strong evidence against the null hypothesis of homogeneity of effects (i.e. p<0.001) the overall relative risk will be considered the most reliable guide to the approximate relative risks in all subgroups.

Analyses will be conducted using STATA® (StataCorp, College Station, Texas, USA) statistical software.

## Conclusions

The results of this IPD meta-analysis will provide a better understanding of the balance of risk and benefits of anti-fibrinolytic treatment in patients with intracranial haemorrhage and how they vary by time to treatment. This knowledge will enable better targeting of the use of anti-fibrinolytics and will influence treatment protocols.
