# Supplementary material for: The effectiveness and safety of anti-fibrinolytics in patients with acute intracranial haemorrhage: statistical analysis plan for an individual patient data meta-analysis
Source: Wellcome Open Res. 2019 Jun 11;2:120. Originally published 2017 Dec 20. [Version 3] doi: 10.12688/wellcomeopenres.13262.3 (PMC5785711; doi:10.12688/wellcomeopenres.13262.3)
Supplement: Peer review history

# Peer review: Reviewer response for version 3

**DOI:** 10.21956/wellcomeopenres.16442.r31010

**Published:** 2019-06-12

**Authors:** Peter A. G. Sandercock

**Affiliations:** 1 Centre for Clinical Brain Sciences, University of Edinburgh, Edinburgh, UK

These amendments seem entirely sensible in view of the accumulating evidence in the field

I have read this submission. I believe that I have an appropriate level of expertise to confirm that it is of an acceptable scientific standard.

# Peer review: Reviewer response for version 1

**DOI:** 10.21956/wellcomeopenres.14388.r29217

**Published:** 2018-01-24

**Authors:** Jonathan Emberson

**Affiliations:** 1 Nuffield Department of Population Health, University of Oxford, Oxford, UK

In my opinion, this statistical analysis plan succinctly describes an appropriate set of analyses for the proposed individual-patient-data meta-analysis. In particular, the approach set out to test and quantify effect modification by treatment delay  (and also age and severity of intracranial bleeding, independently of treatment delay) is appropriate, and offers the best chance of detecting such modification, if it exists. I have no concerns about the methods proposed and look forward to seeing the results. Furthermore, should those results lead to the generation of new hypotheses or further exploratory analyses being done, the methods described in this report should also allow those analyses to be done according to the same general principles/approaches.

I have read this submission. I believe that I have an appropriate level of expertise to confirm that it is of an acceptable scientific standard.

# Peer review: Reviewer response for version 1

**DOI:** 10.21956/wellcomeopenres.14388.r29216

**Published:** 2017-12-21

**Authors:** Peter A. G. Sandercock

**Affiliations:** 1 Centre for Clinical Brain Sciences, University of Edinburgh, Edinburgh, UK

General comment: A guideline on the content of statistical analysis plans has just been published in JAMA by Gamble et al ^1^, with a very useful accompanying Editorial by Demets ^2^.  It would be reasonable for the authors to consider whether they have covered the key points set out in the new guidance document (but interpreted in the light of the comments in the accompanying Editorial); i.e. there is no need slavishly to adhere to the guidance, but rather simply to ensure there are no major omissions in this SAP.

 Specific comments

1. Searches.  The Cochrane Stroke Group’s register of stroke trials would be worth searching separately to Central either via the public portal www.askdoris.org or by contact with the Stroke Group Editorial Base team, since the register contains more information than is downloaded to Central. 
2. Inclusion of studies with > 1000 patients.  This seems sensible.  The authors might in addition consider at least noting the smaller studies identified by the searches and reporting the name, and size of randomised trials with < 1000 patients to give an idea of the totality of evidence (and sadly, an idea of the research waste involved in such small uninformative studies), but not include them in analyses for fear of small study bias etc, etc.
3. Duration of scheduled follow up in CRASH3 and TICH-2 is different; 28 days in CRASH-3 and 90 days in TICH-2.  Some consideration should be given for analyses of death and outcomes around 30 days in both trials to give greater clarity to readers on effects on early outcomes, as well as effects on outcomes at final follow-up.
4. Adjustment for baseline severity.  GCS is measured in CRASH-3, and in TICH-2, the protocol states NIHSS is one of the minimisation variables ^3^ but there is no specific mention of GCS (if GCS is not routinely collected pre-randomisation, the protocol should specify how the NIHSS will be mapped onto GCS or vice versa.).  If TICH-2 does measure GCS pre-randomisation, then it should just be made clear in the SAP.
5. Analyses of neuroradiological outcomes.  These seem reasonable.   

I have read this submission. I believe that I have an appropriate level of expertise to confirm that it is of an acceptable scientific standard.

## References

[1] GambleCKrishanAStockenDLewisSJuszczakEDoréCWilliamsonPRAltmanDGMontgomeryALimPBerlinJSennSDaySBarbachanoYLoderE: Guidelines for the Content of Statistical Analysis Plans in Clinical Trials. *JAMA*.2017;318(23) : 10.1001/jama.2017.18556 2337-2343 10.1001/jama.2017.18556 29260229

[2] DeMetsDLCookTDBuhrKA: Guidelines for Statistical Analysis Plans. *JAMA*.2017;318(23) : 10.1001/jama.2017.18954 2301-2303 10.1001/jama.2017.18954 29260205

[3] SpriggNRobsonKBathPDineenRRobertsIRobinsonTRoffeCWerringDAl-Shahi SalmanRPocockSDuleyLEnglandTWhynesDCicconeALaskaACChristensenHOzturkSCollinsRBereczkiDEgea-GuerreroJJLawZKCzlonkowskaASeiffgeDBeredzieMTICH-2 Investigators: Intravenous tranexamic acid for hyperacute primary intracerebral hemorrhage: Protocol for a randomized, placebo-controlled trial. *Int J Stroke*.11(6) : 10.1177/1747493016641960 683-94 10.1177/1747493016641960 27048694

# Response

**Published:** 2018-02-12

**Authors:** Katharine Ker

**Affiliations:** London School of Hygiene & Tropical Medicine, UK

**Responses to the comments by Peter Sandercock**

 General comment: A guideline on the content of statistical analysis plans has just been published in JAMA by Gamble et al, with a very useful accompanying Editorial by Demets.  It would be reasonable for the authors to consider whether they have covered the key points set out in the new guidance document (but interpreted in the light of the comments in the accompanying Editorial); i.e. there is no need slavishly to adhere to the guidance, but rather simply to ensure there are no major omissions in this SAP.

**Response**

**Thank you for bringing this new article to our attention. We have gone through the checklist of recommended items to check for major omissions and have added detail as appropriate.**

 Searches.  The Cochrane Stroke Group’s register of stroke trials would be worth searching separately to Central either via the public portal www.askdoris.org or by contact with the Stroke Group Editorial Base team, since the register contains more information than is downloaded to Central.

**Response**

**Thank you for this suggestion.  We have added the DORIS database to the search strategy.**

 Inclusion of studies with > 1000 patients.  This seems sensible.  The authors might in addition consider at least noting the smaller studies identified by the searches and reporting the name, and size of randomised trials with < 1000 patients to give an idea of the totality of evidence (and sadly, an idea of the research waste involved in such small uninformative studies), but not include them in analyses for fear of small study bias etc, etc.

**Response**

**We agree.  We will include a description of trials involving <1000 patients. We have inserted text to confirm this.**

 Duration of scheduled follow up in CRASH3 and TICH-2 is different; 28 days in CRASH-3 and 90 days in TICH-2.  Some consideration should be given for analyses of death and outcomes around 30 days in both trials to give greater clarity to readers on effects on early outcomes, as well as effects on outcomes at final follow-up.

**Response**

**We agree with the reviewer.  For our first co-primary outcome we have specified death in hospital within 30 days.  However, as the TICH-2 trial does not collect disability data before 90 days, the second co-primary outcome is death in hospital or dependency at final follow-up within 90 days of randomisation.**

 Adjustment for baseline severity.  GCS is measured in CRASH-3, and in TICH-2, the protocol states NIHSS is one of the minimisation variables but there is no specific mention of GCS (if GCS is not routinely collected pre-randomisation, the protocol should specify how the NIHSS will be mapped onto GCS or vice versa.).  If TICH-2 does measure GCS pre-randomisation, then it should just be made clear in the SAP.

**Response**

**We confirm that the TICH-2 trial collects data on GCS pre-randomisation.  We have added a sentence to the relevant section in the Methods to clarify this.**
